# Description of the Immatures of the Ant, *Myrmelachista catharinae*

**DOI:** 10.1673/031.011.0124

**Published:** 2011-02-25

**Authors:** Daniel Russ Solis, Márcia Akemi Nakano, Eduardo Gonçalves Paterson Fox, Mônica Lanzoni Rossi, Rodrigo Machado Feitosa, Odair Correa Bueno, Maria Santina de Castro Morini

**Affiliations:** ^1^ Centro de Estudos de Insetos Sociais, Instituto de Biociências, São Paulo State University, Postal Code 13506-900, Rio Claro, São Paulo, Brazil; ^2^ Laboratório de Mirmecologia, Universidade de Mogi das Cruzes, Postal Code 08701-970, Mogi das Cruzes, São Paulo, Brasil; ^3^ Laboratório de Histopatologia e Biologia Estrutural de Plantas, Centro de Energia Nuclear na Agricultura, University of São Paulo, Postal Code 13400-970, Piracicaba, São Paulo, Brazil; ^4^ Museu de Zoologia, University of São Paulo, Postal Code 04263-000, Säo Paulo, Säo Paulo, Brazil

**Keywords:** ant, Formicinae, larval instars, Plagiolepidini

## Abstract

The Neotropical ant genus *Myrmelachista* Roger comprises 69 described species and subspecies, and still is a poorly studied group. Larvae play a paramount role in colony nutrition in social hymenopterans and bear considerable value in the reconstruction of group phylogenies, however, they are generally neglected. Larvae of different instars of *Myrmelachista catharinae* Mayr (Hymenoptera: Formicidae) are herein described in detail by light and scanning electron microscopy. The number of larval instars was estimated as three based on the frequency distribution of maximum head capsule widths. The described larvae confirmed some traits typical of the genus: general shape of body and mandibles, general aspect and distribution of body hairs, and the number of sensilla on the palps and galea. Differently from other *Myrmelachista* larvae previously described, *M. catharinae* presented two distinct kinds of second instars, some additional types of body hairs, different number of antennal sensilla, and a distinct labrum shape. *M. catharinae* presented ten pairs of spiracles, which is the first record for this genus.

## Introduction

The ants of the Neotropical genus *Myrmelachista* Roger comprise 69 described species and subspecies (Bolton et al. 2006). These are poorly studied inconspicuous species which dwell in trees, nesting in bark and crevices (Longino 2006).

The importance of larval descriptions to ant systematics and phylogeny has been emphasized and discussed in a number of sources (Wheeler and Wheeler 1976; Schultz and Meier 1995; Pitts et al. 2005; Fox et al. 2007). Of the approximately 12,600 known species of ants (Agosti and Johnson 2010), the larvae of only 7% are presently described and a variable amount of information was given in these descriptions (see Wheeler and Wheeler 1976 and references therein). Among these, the larvae of two species of *Myrmelachista* were partially described by Wheeler and Wheeler (1953).

The present study stands as part of a series of more recent studies on ant larvae aimed at reducing this gap in knowledge. The researchers hereby present a detailed description of each immature stage of the workers of *Myrmelachista catharinae* Mayr (Hymenoptera: Formicidae) with the aid of light and scanning electron microscopy.

## Materials and Methods

The methods described here are similar to those described in previous studies (e.g. Solis et al. 2010b, 2010c), and are repeated here for clarity.

### Obtaining Samples

Nests of *M. catharinae* were obtained in 2008 in the municipality of Mogi das Cruzes (23° 29′ 26.4″ S, 46° 11′ 60.6″ W), São Paulo, Brazil. From these colonies (*n* = 9) preimaginal forms were obtained that were used in the descriptions of *M. catharinae.* It should be noted that the colonies did not present any apparent production of reproductives at the time of collection.

Voucher specimens were deposited in the entomological collection of Laboratorio de Mirmecologia of Universidade de Mogi das Cruzes, São Paulo, Brazil.

### Determining the Number of Larval Instars

As directly following moults from living colonies was impracticable, the number of larval instars was determined using a method detailed in Parra and Haddad (1989). The maximum head widths of the collected larvae (*n* = 132) were measured and the results were plotted on a frequency distribution graph, where every distinct peak suggested a different larval instar. The obtained number of larval instars was tested against Dyar's rule (Parra and Haddad 1989). The first larval instar and the last larval instar could be explicitly identified and thus used as a reference to bracket others.

### Description of the Immature Forms

The morphological descriptions were based on 32 larvae (16 by scanning electron microscopy and 16 by light microscopy), all of which belong to the most frequent head width found for each instar. The larvae were analyzed under a compound light microscope (Zeiss MC80 DX, www.zeiss.com, maximum magnification of 1000 X) and a scanning electron microscope (LEO 435 VP at 20.0 kV). With a stereomicroscope (Zeiss Stemi SV11, maximum magnification of 66X) equipped with a micrometric eyepiece, length and medial width of eggs (n = 5) and larvae (*n* = 63) could be rapidly measured, along with the head width and body length of pupae (*n* = 30).

Terminology used in larval descriptions was based on Wheeler and Wheeler (1976). Data, where applicable, are given as mean ± standard deviation followed by the number (*n*) of observations. The following abbreviations are used: (1) length; (w) width.

All collected samples were fixed in Dietrich's solution (900 ml distilled water, 450 ml 95% ethanol, 150 ml 40% formaldehyde, 30 ml acetic acid) for 24h and then conserved in 70% alcohol. Samples to be analysed under the scanning electron microscope were dehydrated in an alcohol graded series (80–100%; 10 min for each concentration), and critical-point dried (Balzers CPD/030, www.oerlikon.com/balzers/); dried specimens were then attached to aluminium stubs with double-faced conductive adhesive tape and gold-sputtered with a Balzers SCD/050 sputterer. Observations and digital images were made soon after sample preparation. Samples analysed under the compound microscope were prepared by boiling for 5–10 min (depending on the instar) in KOH 10% and then mounted in a small drop of glycerine between glass microscope slides.

## Results

### Determination of Number of Larval Instars

**Figure 1.**
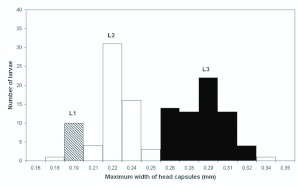
Frequency distribution of the maximum widths of head capsules of *Myrmelachista catharinae* larvae: (L1) first instar, (L2) second instar, and (L3) third instar. The hatched columns represent intervals in which mature embryos in the eggs were found and the black columns represent the interval in which prepupae were found. High quality figures are available online.

The frequency distribution of the width of head capsules resulted in a multimodal distribution with three clear peaks, suggesting the existence of three instars. It was confirmed that the first peak was entirely formed by first instars and the last entirely formed by prepupae (Figure 1). Moreover, the obtained number of larval instars yielded a good fit with Dyar's rule (R^2^ = 0.94). Mean growth rate between the larval instars was 1.24, while between the first and the second instars was 1.21, between the second and third instars was 1.26.

### Morphological Description of the Immature Forms Egg

Ovoid but slightly elongate in shape, with translucent chorion (1 = 0.53-0.60 mm; w = 0.24–0.26 mm; *n* = 5). Length: width ratio 2.28.

### Larvae

As the different instars shared many characteristics, a general description is given first; differences between the different instars are shown in Tables 1–4.

**Figure 2.**
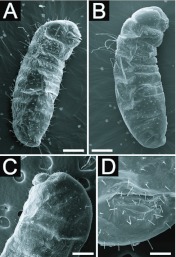
Larvae of *Myrmelachista catharinae:* (A) and (B) side view of second instar; (C) anterior side view of third instar; (D) close on anal region of third instar. Sizes of scale bars: (A) 0.108 mm; (B) 0.100 mm; (C) 0.280 mm; (D) 0.080 mm. High quality figures are available online.

**Figure 3.**
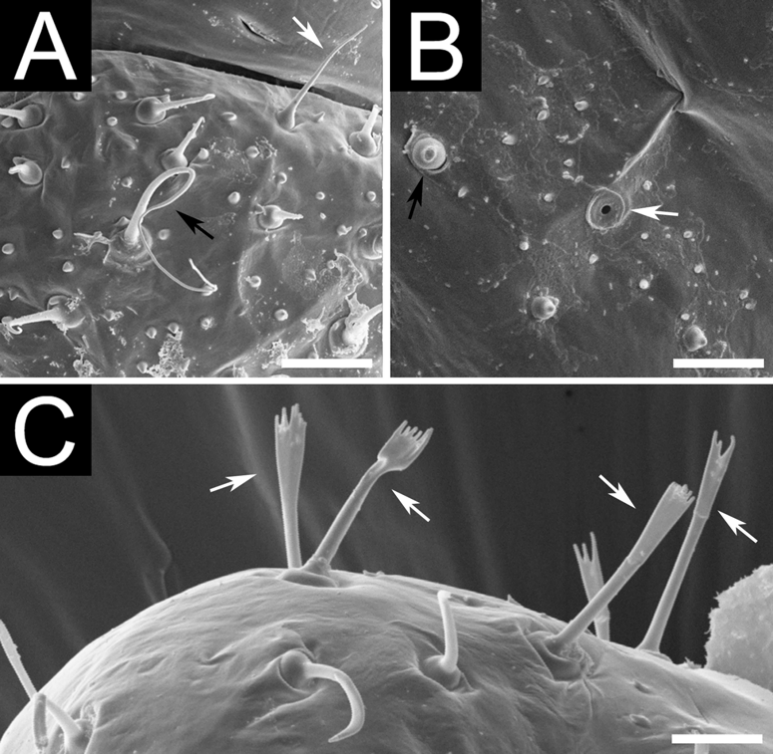
Details of the body integument of second instars of *Myrmelachista catharinae*: a thoracic somite displaying (A) simple hairs of subtype SI (white arrows) and S3 (black arrows) and isolated spinules; (B) another thoracic somite displaying a spiracle (white arrow) and the small elevations resembling basiconic sensilla (black arrow); (C) Subtypes S2 hairs (arrows) by the anus. Sizes of scale bars: (A) 0.016 mm; (B) 0.013 mm; (C) 0.009 mm. High quality figures are available online.

BODY: Body shape ‘crematogastroid’ (Figures 2A, 2B, 2C), which was defined by Wheeler and Wheeler (1976) as “elongate-subelliptical; head applied to ventral surface near anterior end; no neck; somites indistinct”; anus subterminal (Figure 2D). Body integument covered by isolated spinules (Figure 3A). Ten pairs of unornamented spiracles (Figure 3B), being two thoracic and eight abdominal; the two last pairs being slightly smaller than the previous ones which are alike. Types of hairs found are summarized in Table 1, according with analyzed larval instar.

**Figure 4.**
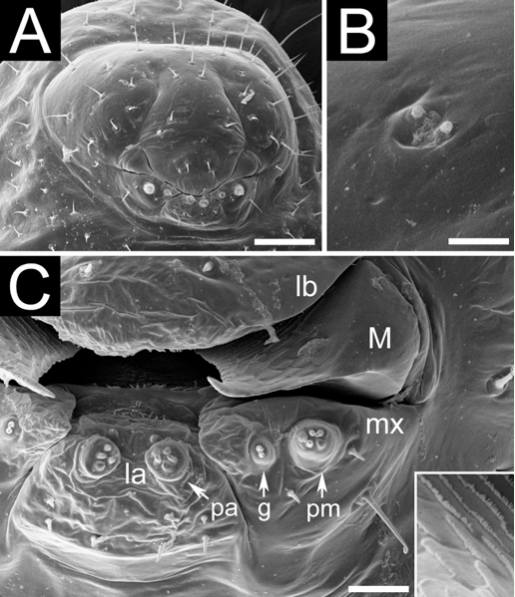
Details on the head capsule of *Myrmelachista catharinae* larvae: (A) full frontal view of a third instar; (B) antenna of a third instar with two sensilla. (C) closer view on the mouthparts of a second instar: labrum (lb), mandible (M), maxilla (mx), galea (g), maxillary palpus (pm), labium (la) and labial palpus (pa). Lower right inlet: surface of the anterior face of the mandibles. Sizes of scale bars: (A) 0.071 mm; (B) 0.007 mm; (C) 0.015 mm. High quality figures are available online.

HEAD CAPSULE (Figure 4A): Subhexagonal; antennae consisting of two or three setaceous sensilla, often arranged in a row, placed over an elliptical elevation (Figure 4B). There is a pair of encapsulated sensilla near the base of each mandible. Clypeus clearly delimited from cranium.

MOUTHPARTS (Figure 4C): Hairs on the mouthparts are always simple, subtype S1 (see Table 1). Labrum subparabolic in shape and partially separated from the ventral border of the clypeus, bearing 4–6 hairs on the anterior face and no sensilla. Mandibles moderately sclerotized and ‘dolichoderoid’ in shape, defined by Wheeler and Wheeler (1976) as “…basal part inflated and narrowed more or less abruptly to the distal part, which is slender and sharp-pointed; no medial teeth or blade”; anterior and posterior surfaces of mandibles with flattened scaly ornamentation. Maxillae conoidal in shape, with 2–3 hairs on its anterior surface and no sensilla; maxillary palps paxilliform with three setaceous sensilla and two basiconic sensilla; galea digitiform with two setaceous sensilla on top. Labium semicircular, surface smooth but with 6–8 hairs and one basiconic sensillum by each extremity of the slit-like spinneret opening. Labial palpus a cluster of three setaceous and two basiconic sensilla. Mouth entrance with hypopharynx endowed with numerous transverse rows of spinules.

### Pupa

During early development pupae are whitish, with eyes and body getting darker later in metamorphosis. Always exarate with no cocoon (body: 1 = 2.67 ± 0.16 mm, 2.40–3.05 mm; head: w = 0.68 ± 0.04 mm, 0.61–0.77 mm; *n* = 30). Only white pupae were measured.

## Discussion

### Determination of number of larval instars

There are estimations of number of larval instars for 64 ant species, of which 22 from four subfamilies presented three larval instars (13 from Formicinae) (Solis et al. 2010a). This is the first estimation for a *Myrmelachista* ant, and it agrees with the range of observations with Formicinae.

### Morphological description of the immature forms

Some of the traits classified as typical of *Myrmelachista* larvae by Wheeler and Wheeler (1953, 1976) were confirmed with this species: general mandible and body shape, types of body hairs (as viewed by optical microscopy, see below), general body hair distribution, and the number of sensilla of the labial and maxillary palps and galea. Unprecedented traits (discussed below) observed with *Myrmelachista* larvae would include: a different morphotype of second instars, observed variations in the types of body hairs in greater magnifications, the presence of ten pairs of spiracles, the number of antennal sensilla, and shape of labrum.

A second instar larva was found in the sample (see Figure 2B) that presented small elevations resembling basiconic sensilla where the subtype S1 hairs should stand (Figure 3B). It was readily discernible for its reduced amount of body hairs. The fact may reflect intraspecific variation, the causes for which can only be guessed at. It might be a reproductive larva, accounting that the samples did not include reproductive pupae or alates. Based on observations with many different species, Wheeler and Wheeler (1976) stated that reproductive larvae usually only differ from worker larvae when mature (i.e. last instar), when they achieve considerably greater bodily dimensions. Edwards and Abraham (1991) observed that workers of *Monomorium pharaonis* Linnaeus kill reproductive larvae while the colony is out of the reproductive phase. Thus the single naked specimen could well be some reproductive larvae that escaped such “communal control”. The matter can only be clarified with direct descriptions of the reproductive larvae of this species, which depend on other samples and/or considerable manipulation of a living colony (e.g. removal of the queen).

Wheeler and Wheeler (1953) described two hair types in larvae of *Myrmelachista,* which correspond to the hair types herein termed S1 and S3. Greater magnification by scanning electron microscope observations revealed structural details that enabled the detection of further hairs types within these two. It should be noted that the Wheelers rarely made use of scanning electron microscope observations, thus could not observe such variations.

The number of antennal sensilla varied between 2–3 for the present species while Wheeler and Wheeler (1953) registered two per antenna and *Myrmelachista ambigua* Forel and *Myrmelachista zeledoni* Emery; they may have failed to observe intraspecific variation due to poor sampling. However, intraspecific variation in the number of antennal sensilla of ant larvae was mentioned by Wheeler and Wheeler (1976), and recently observed by the present authors in other ant species (e.g. Solis et al. 2010c), thus it seems that the phenomenon is widespread.

The main difference observed between the larvae of *M. catharinae* and those of *M. ambigua* and *M. zeledoni* reside in the shape of the labrum, with the labrum of these species being bilobed (Wheeler and Wheeler 1953). Further descriptions within this genus will tell if this interspecific variation holds significant systematic utility.

The information provided herein adds to previous ant larval descriptions, and we hope that it helps guide future investigations on the general biology and taxonomy of ants.

**Table 1.**
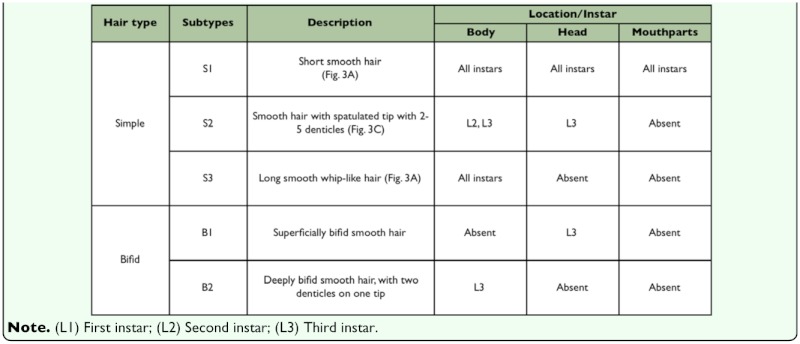
Hair types observed in larvae of *Myrmelachista catharinae.*

**Table 2.**
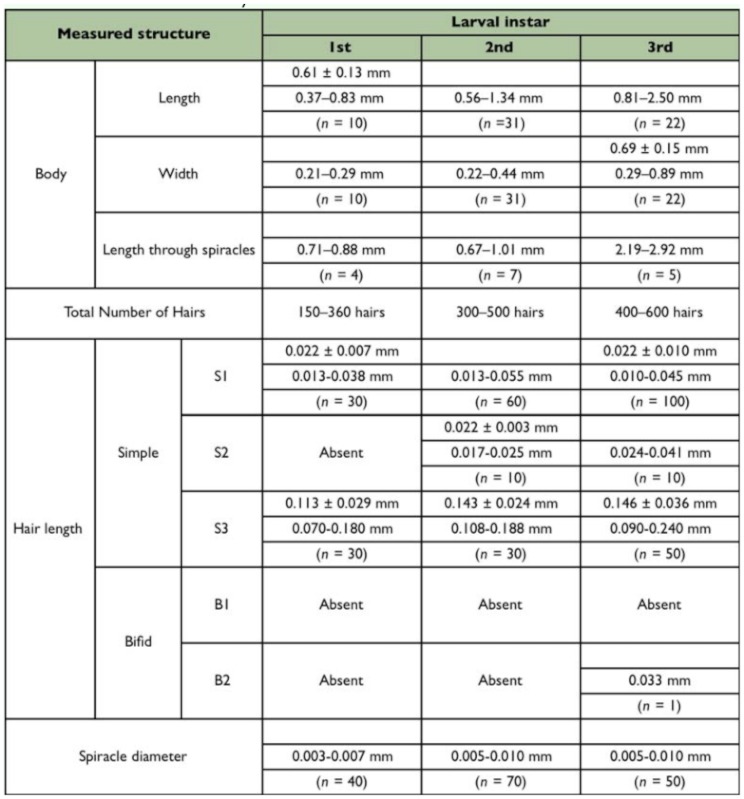
Bodily measures of different instars of *Myrmelachista catharinae.*

**Table 3.**
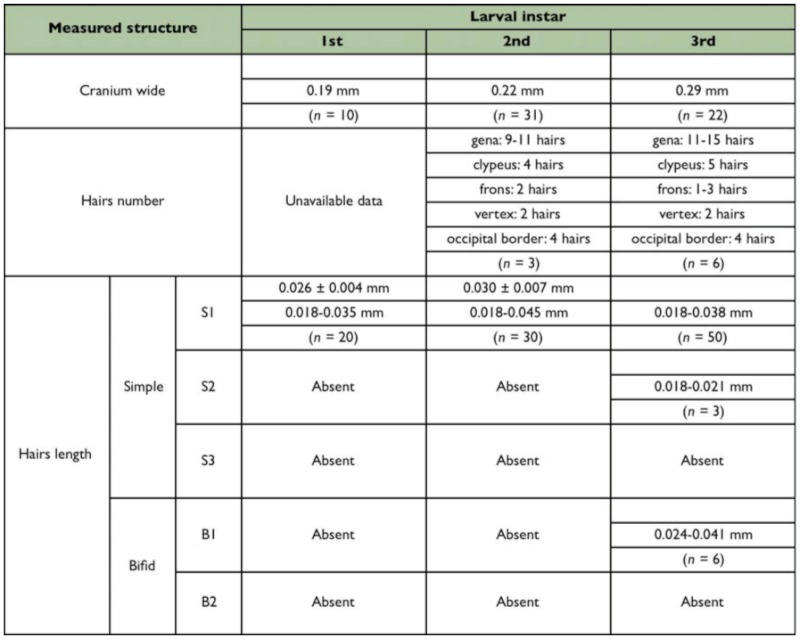
Size of head structures of the three larval instars of *Myrmelachista catharinae.*

**Table 4.**
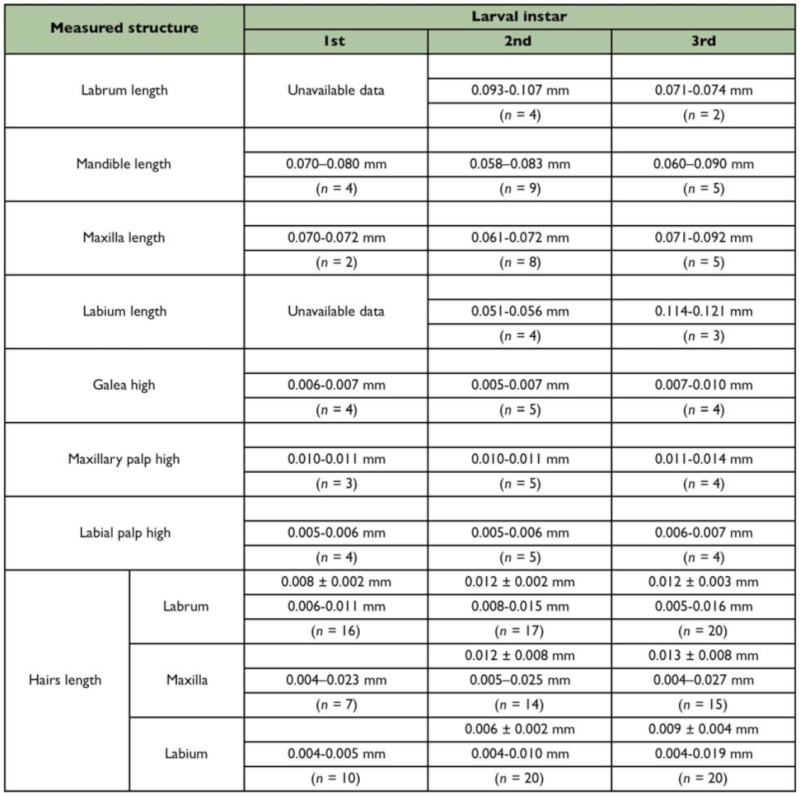
Measures of mouthparts of different instars of *Myrmelachista catharinae.*
